# Cross-country comparison of depressive symptoms and social–emotional aspects in university students from Brazil and Germany during the COVID-19 pandemic: results from two cross-sectional surveys

**DOI:** 10.1192/bjo.2024.762

**Published:** 2024-11-04

**Authors:** Aneliana da Silva Prado, Sabrina Baldofski, Elisabeth Kohls, Alessandra Sant'Anna Bianchi, Fernanda Suemi Oda, Joanneliese de Lucas Freitas, Christine Rummel-Kluge

**Affiliations:** Department of Psychiatry and Psychotherapy, Medical Faculty, Leipzig University, Leipzig, Germany; Department of Psychology, Federal University of Parana, Curitiba, Brazil; and Campus Curitiba, Federal Institute of Education, Science, and Technology of Parana, Curitiba, Brazil; Department of Psychiatry and Psychotherapy, Medical Faculty, Leipzig University, Leipzig, Germany; Department of Psychiatry and Psychotherapy, Medical Faculty, Leipzig University, Leipzig, Germany; and Department of Psychiatry and Psychotherapy, University Leipzig Medical Center, Leipzig, Germany; Department of Psychology, Federal University of Parana, Curitiba, Brazil; Department of Psychology, University of the Pacific, Stockton, CA, USA

**Keywords:** Coronavirus disease (COVID-19), depressive symptoms, mental health, university students, cross-country study

## Abstract

**Background:**

The COVID-19 pandemic negatively affected students’ mental health, increasing pre-existing psychosocial vulnerabilities. University students worldwide have presented differences in their mental health status; however, cross-country studies comparing students’ mental health during the pandemic are lacking.

**Aims:**

To investigate potential differences between university students from Brazil and those from Germany with respect to (a) depressive symptoms and alcohol and drug consumption, (b) social and emotional aspects (loneliness, self-efficacy, perceived stress, social support and resilience) and (c) attitudes towards vaccination.

**Method:**

Two online cross-sectional studies were conducted with university students during the COVID-19 pandemic in Brazil (November 2021 to March 2022) and in Germany (April to May 2022). Depressive symptoms, alcohol consumption, loneliness, self-efficacy, perceived stress, social support, resilience, sociodemographic information and attitudes towards vaccination were assessed. Data were analysed using univariate and bivariate models.

**Results:**

The total sample comprised *N* = 7911 university students, with *n* = 2437 from Brazil and *n* = 5474 from Germany. Brazilian students presented significantly more depressive symptoms and suicidal thoughts, higher levels of perceived stress, higher frequency of drug or substance consumption, and lower levels of perceived social support and resilience than German students, whereas German students presented higher levels of loneliness than Brazilian students. A more favourable opinion towards vaccinations in general was found among Brazilian students compared with German students.

**Conclusions:**

In both countries, low-threshold (online) counselling targeting university students is needed. The differences between the samples could indicate country and/or cultural differences which justify further research in this area.

The COVID-19 pandemic is an unprecedented health crisis in terms of its impact on the whole educational system. As well as the learning process and academic performance, students’ mental health has been affected. For instance, the prevalence of depressive symptoms has increased significantly since the outbreak of the COVID-19 pandemic.^1,2^ The burdens experienced by students have been widely reported, and the need to address youth mental healthcare urgently has been highlighted.^3,4^

## Cross-country studies

Studies comparing the mental health of students from different countries during the COVID-19 pandemic have found differences in anxiety and suicidal thoughts,^5^ depressive and anxiety symptoms,^6^ functional difficulties, stress, and concerns related to COVID-19 pandemic;^7^ studies have also reported stress, anxiety and depression^8^ in university students worldwide, indicating that students as a group are vulnerable to development of mental disorders and aggravation of pre-existing ones. For instance, a comparison between Portuguese and Brazilian university students showed higher levels of depressive symptoms among the latter than among the former.^9^ Compared with university students from Turkey, Poland, Slovenia, Czech Republic, Ukraine, Russia, Israel and Colombia, German students presented a lower prevalence of anxiety symptoms.^6^

Governments’ responses to COVID-19 have had a significant impact on the prevalence of depressive symptoms, with countries where governments implemented stringent policies promptly (according to the Oxford COVID-19 Government Response Index) showing lower depressive symptom prevalence.^10^ This indicates that a rapid public health response, as well as reducing mortality rates, may protect mental well-being and prevent greater psychiatric morbidity by providing coping tools and resilience against uncertainty.^10^ The Oxford COVID-19 Government Response Index is based on 13 metrics, including school closures and workplace closures, restrictions on public gatherings, closures of public transport, stay-at-home requirements, testing policy, face coverings and vaccine policy. The values of this composite measure range from 0 to 100 (100 = strictest). Brazil had a lower index (47.98 to 36.60) than Germany (49.14 to 39.12) throughout the data collection period of this study, except for the period from 11 December 2021 to 31 December 2021.^11^ As there have been few cross-country studies^5–9^ comparing university students’ mental health status in light of their governments’ responses to the COVID-19 pandemic and the changes in study and living conditions that occurred during the pandemic, further research is needed.

## Aims of the study

Brazil and Germany had different governmental responses to the COVID-19 crisis, especially regarding social distancing measures against COVID-19. Germany adopted a more stringent approach, involving stricter restrictions on public gatherings, internal and international travel controls, stay-at-home mandates, face covering requirements and testing policy.^12^ By contrast, Brazil primarily relied on vaccination as its foremost national strategy to mitigate the transmission of COVID-19.^13,14^ Therefore, this study aimed to investigate potential differences between university students from Brazil and from Germany with respect to (a) depressive symptoms and hazardous alcohol use, (b) social and emotional aspects (loneliness, self-efficacy, perceived stress, social support and resilience) and (c) attitudes towards vaccination. The choice of variables was guided by a previous study conducted by our research group,^12^ which identified variations in these variables related to the period of the COVID-19 pandemic.^15,16^ Based on previous results by our research group,^16,17^ we hypothesised that Brazilian university students would show higher levels of depressive symptoms and more difficulties with social and emotional aspects, such as perceived stress, loneliness, social support and resilience. Differences between the Brazilian and German university students’ attitudes towards vaccination were analysed exploratively.

## Method

### Participants and procedures

Data used in this study were obtained from two cross-sectional studies comprising a sample of Brazilian and German university students. Concerning the sample of Brazilian university students, a cross-sectional anonymous online survey was conducted with students at the Federal University of Parana (Brazil) between November 2021 and March 2022 (for more information, see Prado et al^17^). The inclusion criteria were current enrolment as a university student and being 18 years old or older; no exclusion criteria were applied. The ethics committee of the Federal University of Parana granted approval for this study (approval no. 4.625.252, 1 April 2021). The sample comprised *N* = 2437 participants. The Brazilian survey was conducted when no strict social distancing measures were in place in Parana state, and face-to-face academic activities in the university had resumed (on 14 February 2022); although academic and administrative activities were being developed remotely throughout the pandemic period, hybrid activities were allowed at the discretion of each department from September 2021. Vaccination against COVID-19 and wearing a face mask were mandatory to access university buildings. From the beginning to the end of the data collection, the full vaccination of the population in Brazil varied from 54.68 to 74.35%, and the reproduction rate of COVID-19 varied from 0.96 to 0.82.^17^ The Federal University of Parana has around 39 000 students.

Regarding the sample of German university students, a cross-sectional anonymous online survey was conducted with students of six universities (four of which were Universities of Applied Sciences – ‘*Fachhochschule*’ in German), in Saxony, Germany, between April and May 2022 (for more information, see Kohls et al^16^). The inclusion criteria were current enrolment as a university student and being 18 years old or older; again, no exclusion criteria were applied. The ethics committee of the Medical Faculty of Leipzig University granted approval for the current study (file reference: 509/21-ek, 11 November 2021). The sample comprised *N* = 5474 participants. The German survey was conducted when no strict social distancing measures were in place, and face-to-face academic activities had resumed, with face mask mandates applied to university buildings.^11^ From the beginning to the end of the data collection, the full vaccination status of the population in Germany ranged from 73.11 to 75.97%, and the reproduction rate of COVID-19 ranged from 1.20 to 0.81.^11^ Altogether, the German universities had around 69 981 students.

In both countries, participants were recruited using the official email and social media channels of the universities. The survey was set up in the online tool EFS Survey Unipark (version 21.1), a reliable assessment tool which adhered to the data protection guidelines in both countries. All participants provided online informed consent before participating via an opt-in function, where they were informed about the voluntary nature of participation and the guarantee of anonymity.

### Measures

This study draws on data and results from two congruent cross-sectional surveys conducted in Brazil^10^ and Germany^11^ that used identical questionnaires. The ENRICHD Social Support Instrument (ESSI) and the question on attitude towards vaccination were translated to Portuguese using a back-translation method. Further information on the psychometric properties of the instruments (including Cronbach's alpha) can be found in our previous publications.^16,17^

### Sociodemographic information, physical conditions and vaccination status

In both surveys, the following sociodemographic data were assessed: gender (female, male, diverse), age, relationship status (in a relationship or single), being a parent, residential status (living alone or with other people), study programme (bachelor, master or other) and migration background (self, parents or no migration background). In addition, chronic somatic diseases (‘Do you suffer from a (chronic) physical illness?’) and diagnosed mental disorders were assessed with ‘yes’ or ‘no’ answer options. Chronic somatic diseases were assessed because people with such conditions were at greater risk of dying from COVID-19 or suffering from long-term effects of COVID-19. COVID-19 vaccination status (‘What is your current COVID-19 vaccination status?’) was also assessed. Given the differences in the studies conducted in Germany and Brazil and the respective evolving COVID-19 vaccination scenarios, the answer options differed. For the Brazilian sample, they were ‘I am partly vaccinated (one shot)’, ‘I am fully vaccinated (two shots or single shot)’ and ‘I am not vaccinated’. This was because the Federal University of Parana required students to be fully vaccinated to be allowed to be present in face-to-face classes when they resumed; this did not occur in the German universities where the study took place. For the German sample, the answer options encompassed ‘I am fully vaccinated’, ‘I am not fully vaccinated, but would like to be vaccinated’ and ‘I am not fully vaccinated and do not want to be vaccinated’. The data were dichotomised into ‘fully vaccinated’ and ‘not vaccinated’, and Brazilian participants who reported being partly vaccinated (*n* = 40, 1.6%) were excluded from the dichotomisation.

### Depressive symptoms

Depressive symptoms over the past 14 days were assessed using the Patient Health Questionnaire-9 (PHQ-9).^18,19^ The PHQ-9 is a validated and widely used instrument for assessment of depression severity and comprises nine items on a four-point Likert scale from 0 = ‘not at all’ to 3 = ‘nearly every day’.^18^ The sum score ranges from 0 to 27, with higher scores indicating higher levels of depressive symptoms. A sum score of 10 or more indicates clinically relevant depressive symptoms (‘moderate to severe’).^18^ Item 9 (‘thoughts that you would be better off dead, or of hurting yourself’) indicates suicidal thoughts when answered with a score of ≥1 (i.e. reporting suicidal thoughts on several days or more during the past 14 days).

### Alcohol and drug consumption

Levels of alcohol consumption were assessed with the hazardous use subscale of the Alcohol Use Disorders Identification Test (AUDIT-C).^20^ With a five-point Likert scale ranging from 0 = ‘never’ to 4 = ‘four or more times a week,’ the subscale assesses the frequency of drinking alcohol (‘How often do you have a drink containing alcohol?’), the typical quantity of alcohol consumed (0 = ‘one or two’ up to 4 = ‘ten or more’) and the frequency of consuming large quantities of alcohol (i.e. six or more drinks on one occasion; 0 = ‘never’ to 4 = ‘once a week’). The total hazardous use subscale sum score ranges from 0 to 12, with higher scores indicating higher alcohol consumption and related risk.

One item of the AUDIT-C was rephrased to ‘drug or substance use’ (0 = ‘never’ to 4 = ‘four or more times a week’) and was used to assess the frequency of drug consumption.

### Social and emotional aspects

Experience of loneliness was assessed using the UCLA three-item loneliness scale.^21,22^ Each item was answered on a four-point Likert scale ranging from 0 = ‘never’ to 3 = ‘often’, with a total sum score ranging from 0 to 9. Higher scores indicate more experience of loneliness.

A general sense of perceived self-efficacy was assessed with the ten-item General Self-Efficacy Scale (GSE).^23,24^ Items were rated on a four-point Likert scale ranging from 1 = ‘not at all true’ to 4 = ‘exactly true’, with a sum score ranging from 10 to 40. Higher values indicate higher levels of self-efficacy.

Perceived stress was assessed using the Perceived Stress Scale (PSS-4),^25,26^ which has four items on a five-point Likert scale ranging from 0 = ‘never’ to 4 = ‘very often’. The total sum score ranges from 0 to 16, with higher scores indicating more perceived stress.

Social support was assessed with the ESSI.^27^ The five items were rated on a five-point Likert scale from 1 = ‘none of the time’ to 5 = ‘all of the time,’ with a total sum score ranging from 5 to 25. Higher scores indicate higher levels of social support.

The Brief Resilience Scale (BRS)^28,29^ was used to measure the ability to bounce back or adapt well in the face of adversity (e.g. ‘I tend to bounce back quickly after hard times.’), with a five-point Likert scale ranging from 1 = ‘strongly disagree’ to 5 = ‘strongly agree’. The total sum score ranged from 1 to 5. Higher values indicate higher levels of resilience.

### Attitude towards vaccination

Participants in both samples were asked to rate their attitudes towards vaccination in general (‘What is your attitude towards vaccinations in general?’) on a five-point Likert scale from 1 = ‘rejecting’ to 5 = ‘supporting’.

### Statistical analysis

Descriptive statistics on sociodemographic characteristics, depressive symptoms, social and emotional aspects, and attitudes towards vaccination in both samples (Brazilian and German university students) are reported. Chi-squared tests were performed to estimate the differences between the samples with respect to the following categorical variables: gender, relationship status, being a parent, residential status, study programme, migration background, clinically relevant depressive symptoms and suicidal thoughts. Where needed, significant effects found using χ^2^-tests were further decomposed using a *z*-test to compare column proportions. For group comparisons of the continuous dependent variable age, a *t*-test was performed.

Separate one-way analyses of covariance (ANCOVAs) were performed to estimate the differences between Brazilian and German university students with respect to the continuous dependent variables (depressive symptoms, alcohol use, loneliness, social support, self-efficacy and attitude toward vaccinations). The mean scores were controlled for age and gender as covariates in all analyses. A bootstrapping procedure (1000 resamplings; 95% bias-corrected and accelerated confidence intervals (CI BCa)) was used to correct for group size differences and deviations from a normal distribution and to present 95% confidence intervals for the means. Bonferroni correction was applied to adjust for multiple testing when applicable. To estimate effect sizes for chi-squared tests, the ϕ coefficient was used; Cramér's *V* (ϕ_c_) was used when the contingency table was larger than 2 × 2, with ϕ, ϕ_c_ = 0.10 indicating a small effect, ϕ, ϕ_c_ = 0.30 an average effect and ϕ, ϕ_c_ = 0.50 a large effect.^30^ The effect size was interpreted as small when *d* < 0.20, medium when *d* < 0.50 and large when *d* > 0.50 for the *t*-test; whereas *η*²partial = 0.001 was interpreted as small, *η*²partial = 0.06 as medium and *η*²partial = 0.14 as large for the ANCOVAs.^30^

The analyses of sociodemographic data were used to present sample characteristics (i.e. analysis of potential sociodemographic differences between the samples of German and Brazilian students), whereas the analyses of psychosocial, emotional and behavioural outcomes were conducted to answer the research questions. All statistical analyses were performed using IBM SPSS Statistics version 27.0. A two-tailed α = 0.05 was applied to statistical testing.

### Ethics statement

The Federal University of Parana's ethics committee (approval no. 4.625.252, 1 April 2021) and the Medical Faculty of Leipzig University's ethics committee (file reference: 509/21-ek, 11 November 2021) granted approval for the current study. All participants provided informed consent before participation via an online opt-in function. This manuscript is an honest, accurate and transparent account of the study being reported, and no important aspects of the study have been omitted.

## Results

The sociodemographic characteristics of both samples are presented in Table 1. There were statistically significant differences between Brazilian and German students for all sociodemographic variables. Specifically, German students were significantly more likely than Brazilian students to be female, single, not a parent, living alone and enrolled in a master's programme and to have a migration background. Brazilian students were older [mean deviation (*MD*) = 4.132, *P* < 0.001, 95% CI BCa (3.773, 4.516)], with ages ranging from 18 to 71 years (*M* = 27.84, 95% CI BCa 27.49–28.22), compared with German students, whose ages ranged from 18 to 51 years old (*M* = 23.71, 95% CI BCa (23.56, 23.85). Age was the only variable showing a large effect size (*d* = 0.67).

**Table 1:** Sample characteristics and group differences in sociodemographic information and health status (*N* = 7911)

Variable, *n* (%)^a^	Brazilian students	German students	Test	*P*-value	Effect size
	(*n* = 2437)	(*n* = 5474)			
Gender, *n* (%)			χ^2^(2,7911) = 16.313	**<0.001**	ϕ_c_ = 0.045
Female	1579 (64.8)*	3775 (69.0)*			
Male	821 (33.7)*	1599 (29.2)*			
Diverse	37 (1.5)	100 (1.8)			
Age, *M* (s.d.)	27.84 (8.41)	23.71 (4.81)	*t*(3167.323) *=* 22.659	**<0.001**	*d* = 0.672
Relationship status, *n* (%)			χ^2^(1,7911) = 29.527	**<0.001**	ϕ = 0.061
In a relationship	1349 (55.4)	2668 (48.7)			
Single	1088 (44.6)	2806 (51.3)			
Being a parent, *n* (%)	335 (13.7)	316 (5.8)	χ^2^(1,7911) = 141.968	**<0.001**	ϕ = 0.134
Residential status, *n* (%)			χ^2^(1,7911) = 137.505	**<0.001**	ϕ = 0.132
Alone	319 (13.1)	1355 (24.8)			
Not alone	2118 (86.9)	4119 (75.2)			
Study programme, *n* (%)			χ^2^(2,7911) = 936.057	**<0.001**	ϕ_c_ = 0.344
Bachelor	1404 (57.6)*	1928 (35.2)*			
Master	506 (20.8)*	3108 (56.8)*			
Other	527 (21.6)*	438 (8.0)*			
Migration background, *n* (%)			χ^2^(2,7911) = 216.666	**<0.001**	ϕ_c_ = 0.165
Self	28 (1.1)*	338 (6.2)*			
Parents	27 (1.1)*	362 (6.6)*			
None	2437 (97.7)*	4774 (87.2)*			
Diagnosed mental disorder	887 (36.5)	1239 (22.6)	χ^2^(1,7906) = 164.008	**<0.001**	ϕ = 0.144
Chronic somatic diseases	383 (15.8)	933 (17.0)	χ^2^(1,7895) = 1.811	0.178	ϕ = 0.178
Fully vaccinated against COVID-19	2372 (97.3)	5102 (93.2)	χ^2^(1, 7911) = 55.075	**<0.001**	ϕ = 0.083

Bold font indicates statistical significance.

a. Percentages were calculated based on valid cases.

Further, Brazilian students were more likely to have a diagnosed mental disorder than German students (*P* < 0.001). The most commonly diagnosed mental disorders reported by Brazilian students were anxiety (*n* = 568, 35.2%), depression (*n* = 433, 26.8%), other mental disorders (*n* = 157, 9.7%) and attention-deficit hyperactivity disorder (*n* = 118, 7.3%). German students reported depression (*n* = 612, 31.1%), anxiety (*n* = 437, 22.2%), other mental disorders (*n* = 333, 16.8%) and eating disorders (*n* = 215, 10.9%) as the most commonly diagnosed mental disorders. Finally, Brazilian students were more likely to be fully vaccinated against COVID-19 than German students (*P* < 0.001).

### Depressive symptoms

There was a statistically significant difference between Brazilian and German students, with the former presenting higher levels of depressive symptoms than the latter [*MD* = 4.943, 95% CI Bca (4.595, 5.296)] (Table 2). The covariates age and gender had significant effects on the PHQ-9 sum scores model (both *P* < 0001; *η*^2^partial = 0.010 and *η*^2^partial = 0.001, respectively). Further, significantly more Brazilian students reported clinically relevant depressive symptoms [95% CI BCa (−0.260, −0.219)], and they had a higher prevalence of suicidal thoughts [95% CI BCa (−0.170, −0.122)] compared with German students (Table 2).

**Table 2:** Means, standard deviations and confidence intervals for mental health measures and social and emotional characteristics of Brazilian and German samples during COVID-19 (*N* = 7911)

Variable	Brazilian students		German students		Test	*P*-value	*η*^2^partial/ϕ
	(*n* = 2437)		(*n* = 5474)				
	*M*^a^ (s.d.)	95% CI	*M*^a^ (s.d.)	95% CI			
Depressive symptoms (PHQ-9)	12.85 (7.40)	12.55–13.15	8.35 (5.52)	8.20–8.49	*F*(1, 7907) = 1002.293	<0.001	*η*^2^ = 0.112
Alcohol use (AUDIT-C)	2.80 (2.52)^b^	2.70–2.91	2.91 (2.31)	2.27–2.35	*F*(1, 7891) = 2.912	0.088	*η*^2^ = 0.000
Drug or substance consumption	2.13 (1.28)	1.99, 2.28	1.74 (1.03)	1.67, 1.81	*F*(1, 1097) = 18.940	<0.001	*η*^2^ = 0.017
Loneliness (UCLA-3)	5.25 (2.35)	5.16–5.34	5.75 (1.86)	5.70–5.80	*F*(1, 7907) = 37.848	<0.001	*η*^2^ = 0.005
Self-efficacy (GSE)	2.82 (0.59)	2.80–2.85	2.77 (0.46)	2.76–2.78	*F*(1, 7907) = 0.077	0.782	*η*^2^ = 0.000
Perceived stress (PSS-4)	7.81 (2.52)^c^	7.70–7.92	7.23 (3.20)	7.15–7.31	*F*(1, 7179) = 60.924	<0.001	*η*^2^ = 0.008
Social support (ESSI)	18.04 (5.15)	17.93–18.25	20.71 (4.10)	20.60–20.83	*F*(1, 7907) = 539.367	<0.001	*η*^2^ = 0.064
Resilience (BRS)	2.75 (0.87)^d^	2.72–2.79	3.04 (0.77)	3.02–3.06	*F*(1, 864) = 270.633	<0.001	*η*^2^ = 0.033
Attitude towards vaccination	4.92 (0.44)	4.90 (4.93)	4.63 (0.78)	4.61–4.65	*F*(1, 7840) = 270.435	<0.001	*η*^2^ = 0.033
	*n* (%)		*n* (%)				
Suicidal thoughts^e^	808 (33.2)	NA	1075 (19.6)	NA	χ^2^(1, 7911) = 169.880	<0.001	ϕ = 0.147
Clinically relevant depressive symptoms^f^	1488 (61.1)	NA	1936 (35.4)	NA	χ^2^(1, 7911) = 453.399	<0.001	ϕ = 0.239

PHQ-9, Patient Health Questionnaire-9; AUDIT-C, Alcohol Hazardous Use Subscale of the Alcohol Use Disorders Identification Test; ESSI, ENRICHD Social Support Instrument; GSE, General Self-Efficacy Scale; PSS-4, Perceived Stress Scale; UCLA, Three-Item Loneliness Scale. NA, not applicable.

a. Scores adjusted for age and gender; bootstrapping sample.

b. Reduced sample size of *n* = 2421 participants owing to missing data.

c. Reduced sample size of *n* = 1709 participants owing to missing data.

d. Reduced sample size of *n* = 2394 participants owing to missing data.

e. Suicidal thoughts based on item 9 score ≥1 on PHQ-9.

f. Clinically relevant depressive symptoms based on having a score of 10 or more on the PHQ-9.

### Alcohol and drug or substance consumption

Regarding hazardous alcohol use (hazardous subscale of AUDIT-C), there was no statistically significant difference between Brazilian and German students (*P* = 0.088). The covariate gender presented a significant effect on the model (*P* < 0.001; *η*^2^partial = 0.022), whereas age did not (*P* = 0.053).

Concerning drug or substance consumption, *n* = 2074 (85.1%) of Brazilian participants reported having never consumed any drug or substance, as did *n* = 4720 (86.2%) of German participants, with no statistically significant difference found between the two samples [χ^2^(1, 7911) = 1.748, *P* = 0.186]. Among those who reported any drug consumption, Brazilian students had a higher frequency of drug or substance consumption than German participants [*MD* = 0.323, 95% CI BCa (0.154, 0.507)]. Both covariates, age and gender, had significant effects on the model (both *P* < 0.05, *η*^2^partial = 0.016 and *η*^2^partial = 0.007, respectively).

### Social and emotional aspects

German students presented significantly higher levels of loneliness (UCLA-3) compared with Brazilian students [*MD* = 0.315, 95% CI Bca (0.203, 0.423)]. The covariate age had a significant effect on the model (*P* < 0001; *η*^2^partial = 0.018), whereas gender did not (*P* = 0.508). Self-efficacy (GSE) sum scores did not differ between Brazilian and German students (*P* = 0.782). Age and gender had significant effects on the model (both *P* < 0001; *η*^2^partial = 0.020 and *η*^2^partial = 0.004, respectively). Brazilian students had significantly higher sum scores for perceived stress (PSS-4) than German students [*MD* = 0.692, 95% CI Bca (0.532, 0.851)]. Age and gender had significant effects on the model (both *P* < 0001; *η*^2^partial = 0.002 and *η*^2^partial = 0.003, respectively). Social support (ESSI) sum scores were significantly higher among German students compared with Brazilian students [*MD* = 2.611, 95% CI Bca (2.391, 2.832)]. Gender had a significant effect on the model (*P* < 0001; *η*^2^partial = 0.018), but age did not (*P* = 0.711). Finally, resilience (BRS) sum scores were significantly higher among German students compared with Brazilian students [*MD* = 0.336, 95% CI Bca (0.297, 0.373)]. Both covariates, age and gender, had significant effects on the model (both *P* < 0001; *η*^2^partial = 0.006 and *η*^2^partial = 0.014, respectively).

### Attitude towards vaccination

Brazilian students presented statistically significantly more favourable attitudes towards vaccination in general compared with German students [*MD* = 0.290, 95% CI BCa (0.261, 0.318)]. Neither of the covariates (age or gender) had a significant effect on the model (both *P* > 0.05).

## Discussion

This study investigated differences between university students from Brazil and Germany regarding mental health outcomes, social and emotional aspects, and attitudes towards vaccination. As hypothesised, Brazilian students reported more depressive symptoms and suicidal thoughts than German students. Despite the between-sample differences in sociodemographic characteristics, we considered our Brazilian and German groups to be representative of their respective university student populations, with differences related to each country's geopolitical and cultural characteristics. The sample sizes were substantial, and the results for each country were comparable with findings of similar studies conducted in both countries.^16,17^ However, the diversity within each country should be considered when evaluating the results and the extent to which they can be generalised, as in both samples the universities were located in one region/federal state.

In Brazil, a Latin American middle-income country, there was a compartmentalised response to the COVID-19 crisis, and each subnational government managed its own health policies, with an important role for governors, with little or no support from the federal government – which eventually produced horizontal and vertical competition for scarce supplies in the fight against COVID-19.^31,32^ Therefore, social distancing measures, use of masks and testing could vary significantly across the country.

Germany, a European high-income country, implemented a mitigation strategy to curb the spread of COVID-19. Lockdowns and strict rules regarding social distancing were imposed nationwide at different points in time.^11,16^ Although these restrictions were alternated with relaxation of control measures, face masks were mandatory at different time points across German regions.^33^ Germany was also among the countries that implemented mandatory COVID-19 certification for proof of vaccination status (at least two shots) to enter restaurants, stores, nightclubs and gyms, among other public services, and also to travel internationally.^34^

In both Germany and Brazil, similar to other countries, misinformation regarding COVID-19 was an important issue that may have negatively affected prevention measures.^7,35^ On the other hand, trustworthy sources of information may positively affect how people respond psychologically and behaviourally to crises.^36^ Accordingly, an immediate and consistent response can be considered a powerful public health prevention tool for health crises such as the COVID-19 pandemic worldwide.

### Depressive symptoms

Considering the sample as a whole, *n* = 3424 (43.3%) participants reported clinically relevant depressive symptoms. Although these results do not represent clinical diagnoses, as PHQ-9 is a self-report instrument, they corroborate previous literature indicating that university students worldwide are at increased risk of developing depression.^1–4,15,37^

Brazilian students reported higher levels of depressive symptoms and had a greater prevalence of clinically relevant depressive symptoms and suicidal thoughts than German students. The effect sizes observed between the two groups indicated a higher risk among Brazilian students of developing depression compared with German students. The prevalence of clinically relevant depressive symptoms among Brazilian students in our sample was higher compared with those reported in France (16.1%),^38^ the USA (48.14%),^39,40^ China (22.0%) and Ethiopia (46.3%)^41^ and in the Brazilian general population (41.9%).^42^ The prevalence of clinically relevant depressive symptoms among German students was similar to the pooled prevalence (34%) of depressive symptoms among university students during the pandemic worldwide.^1,2^ Before the pandemic, prevalences of 6.1 to 65.5% for depressive symptoms and 3.9 to 49.1% for suicidal ideation among university students internationally were reported.^3^ An increased risk of suicide among university students during the COVID-19 pandemic has been reported in both Brazil^43^ and Germany,^16^ as well as higher numbers of students reporting suicidal thoughts in both countries. Our results may be related to the higher likelihood of Brazilian students having received a diagnosis of any mental disorder (36.5%) compared with German students (22.6%), as this has been found to be a predictor of depressive symptoms among university students.^16,17^

By contrast, although German students had a higher prevalence of diagnosed depression disorder (31.1%) compared with Brazilian students (26.8%), their levels of self-reported depressive symptoms and suicidal thoughts were lower than those of their Brazilian counterparts. Considering that 47.7% (*n* = 591) of the German students who disclosed having been diagnosed with any mental disorder were receiving any form of treatment (i.e. psychotherapy and/or medication),^16^ compared with 63.6% (*n* = 564) of the Brazilians,^17^ we hypothesise that this could be pandemic-related, as previous research has indicated that countries where governments implemented stringent policies promptly (which was the case in Germany) had lower prevalence of clinically significant depressive symptoms.^10^ As we assessed lifetime diagnoses, some participants may have no longer been in need of treatment by the time the survey was conducted. In addition, the high number of deaths by COVID-19 in Brazil may indicate that Brazilian students experienced grief more frequently; this could be related to the prevalence of depressive symptoms and suicidal thoughts in this sample. On the other hand, bereavement-related emotions are often mistaken for symptoms of major depressive disorder.^44^ Longitudinal studies could help us to understand this phenomenon better.

### Alcohol and drug or substance consumption

Although levels of hazardous alcohol use did not differ between the two groups, a higher frequency of drug or substance consumption was observed among Brazilian students compared with German students. Changes in alcohol consumption among university students during the COVID-19 pandemic have been reported, with studies finding that it increased or decreased, although some studies reported no changes.^15,45–47^ There have also been heterogeneous results regarding drug or substance consumption during COVID-19. Increases in drug or substance use during the COVID-19 pandemic have been reported worldwide,^48^ as have decreases or no changes.^49^ Returning to live with family was associated with less alcohol consumption.^45^ Both alcohol and drug or substance use are associated with poorer mental health outcomes, such as depressive symptoms, and social and emotional aspects, such as boredom.^48,49^ Nevertheless, attributing causes to the differences between the two samples would require more comprehensive measures and longitudinal studies.

### Social and emotional aspects

Although German students experienced higher levels of perceived loneliness than Brazilian students, they also reported higher levels of perceived social support. The higher levels of loneliness among German students could be related to their sociodemographic characteristics, as they were more likely to be single, living alone and not a parent compared with Brazilian students. These higher levels of loneliness could also be related to the more frequent and extended periods of lockdowns in Germany compared with Brazil. Although loneliness has been identified as a predictor of suicidal ideation and behavior^50^ and associated with more depressive symptoms,^51^ we believe that social support worked as a protective factor against the impact of loneliness on mental health outcomes^52^ among German students.

Similarly, the level of perceived loneliness among Brazilians could be understood in light of the sociodemographic characteristics of that sample (i.e. living with others, having a relationship, being a parent) and the more relaxed social restriction measures applied in Brazil compared with Germany. However, availability of social support indicates the extent to which an individual perceives that they can rely on their social relationships and feels valued and cared about.^27^ This means that one may feel connected but not supported. Therefore, the lower levels of perceived social support among Brazilians despite the lower levels of loneliness compared with Germans could be related to the higher levels of depressive symptoms^51^ in the Brazilian sample (e.g. helplessness). Moreover, it could reflect aspects of the culture and the psychosocial implications of socioeconomic inequality, which increased during the COVID-19 pandemic, affecting interpersonal relations, domestic and academic life, and work–life balance.^9,37,42^ Finally, these results illustrate the relevance of assessing both loneliness and social support constructs.^52^

In both samples, perceived social support was found to be a joint protective factor for resilience and self-efficacy.^16,17^ Although self-efficacy did not differ between the two samples, German students reported higher resilience levels compared with Brazilian students. As social support and resilience have been identified as protective factors against depressive symptoms^12^ and suicidal thoughts,^5^ these results could also reflect the differences observed in depressive symptoms reported above, as less perceived satisfactory support is associated with more suicidal thoughts among university students.^5^

The higher levels of perceived stress among Brazilian students compared with German students may be related to their government's response to the COVID-19 crisis. The denial of the seriousness of the pandemic by the Brazilian Federal Government and its late response to the COVID-19 crisis led Brazil to become one of the countries with the highest number of deaths from COVID-19 worldwide.^31,32^ Moreover, Brazil's political instability, social inequality and insecurity may affect students directly. For some Brazilian students, the shift to online teaching became a barrier to study continuation owing to lack of or poor equipment or internet access. German students also experienced negative effects of the pandemic and faced changes in their study and living conditions that included a shift to remote teaching, lack of social contact with other students and financial loss.^12,15^ They were more likely to report being worried and thinking about the coronavirus than the German general population^35^ and showed an increased prevalence of suicidal ideation compared with 2020 and 2021,^35^ indicating long-term effects of the pandemic.

### Attitude towards vaccination

A more favourable opinion towards vaccinations, in general, was found among Brazilian students compared with German students. This is in line with the higher likelihood of Brazilian participants to be fully vaccinated against COVID-19 (i.e. 97.3%) compared with their German counterparts [93.2%; χ^2^(1, 7911) = 55.075, *P* < 0.001, ϕ = 0.083]. Unlike the Brazilian university where the survey took place, in German universities, a full vaccination certificate was not required to access the university; this may have influenced the number of fully vaccinated students. Moreover, the survey was conducted earlier in Brazil than in Germany, and the percentages of the populations that were fully vaccinated were 74.35% in Brazil and 75.97% in Germany.^11^

Brazil has a structured immunisation strategy within its public universal healthcare system^53^ and rapidly increased vaccination against COVID-19 as soon as the vaccine was authorised in the country.^14^ In Germany, vaccination is mostly provided by physicians in private practices. When a vaccine is officially recommended in the national immunisation schedule, the costs of childhood and adult vaccination are fully reimbursed by health insurance funds. Vaccination hesitancy varies across countries,^14^ and more favourable opinions towards vaccinations in general have been reported to predict vaccination intention.^35^ From a public health perspective, this implies a need to design vaccination campaigns that address contextual barriers and negative beliefs toward vaccination.

### Strengths and limitations

This study had several strengths. First, it had a large sample size that we considered to be representative of the respective countries. Second, we used identical measures for the data collection in both countries, ensuring the comparability of results. Third, we used validated, internationally comparable and widely used questionnaires. The study also had some limitations. First, owing to our exploratory approach and the nature of the data, we did not consider further hypotheses regarding different cultural aspects that might have influenced sociodemographic differences. Specifically, there was a statistically significant difference in age between the two samples, with German students being younger than Brazilian students. By contrast, Brazilian students were more likely to be doing a bachelor degree. This difference might be related to later access to university in Brazil compared with Germany, probably owing to educational differences. We addressed this limitation by controlling for age as a covariate in all analyses. Second, we used one item for screening illicit drug use, which was rephrased from the AUDIT-C questionnaire, and analysed the results descriptively. We acknowledge the limitation of not using a standardised measure for illicit substance use and suggest this could be included in future studies. Third, we assessed attitudes toward vaccination with a single item, which was analysed descriptively to characterise the samples according to the COVID-19 context in each country; future research could survey vaccination attitudes in more detail and evaluate how sociodemographic characteristics (including country of origin) and psychosocial and emotional aspects could affect attitudes towards vaccination. Further, the two time points of data collection were not identical, although they were very close; this should be considered when analysing the results. Nevertheless, regardless of time point, Brazil and Germany had different government measures regarding social distancing. Our results shed light on possible long-lasting effects of the COVID-19 pandemic on university students’ mental health and indicate a need to longitudinally assess students’ mental health status and provide mental healthcare tailored to them.

### Future implications

This study illustrates the negative impact of the COVID-19 pandemic on the mental health of university students in Brazil and Germany and highlights difficulties in coping, high levels of social and emotional distress and clinically relevant symptoms among students, in line with international findings.^1–8,12,15–17,35,37–39,41,43,46^ Although the results were obtained using self-report screening instruments and not clinical assessment, they strongly indicate a need for mental health promotion strategies to prevent maladaptive coping mechanisms such as alcohol and drug use^15^ and prevent mental disorders from developing and becoming chronic.^10^ Although fear, grief and anxiety are expected responses to such a health crisis,^36^ they also indicate that regardless of the country, mental healthcare should be widely and easily accessible to university students, together with sanitary measures to control a pandemic. The differences between the samples could indicate country/cultural differences that could be further investigated and should be considered when managing a global health emergency such as the COVID-19 pandemic. Finally, the study highlights that university students are at increased risk of developing mental disorders in times of crises, underlining a critical call to action for universities to proactively enhance support systems (for instance, through online support groups^54^). Thus, it seems likely that providing mental health promotion programmes and implementing accessible, low-threshold (online or in-person) mental health support targeting university students may help them to cope.

## Supporting information

Prado et al. supplementary material Prado et al. supplementary material

## Data Availability

The data are not publicly available owing to ethical restrictions. However, the data that support the findings of this study are available from the corresponding author (A.d.S.P.) upon reasonable request.
